# Transgressive Biochemical Response to Water Stress in Interspecific Eggplant Hybrids

**DOI:** 10.3390/plants12010194

**Published:** 2023-01-03

**Authors:** Sara González-Orenga, Mariola Plazas, Elvira Ribera, Claudia Pallotti, Monica Boscaiu, Jaime Prohens, Oscar Vicente, Ana Fita

**Affiliations:** 1 Institute for the Conservation and Improvement of Valencian Agrodiversity (COMAV), Universitat Politècnica de València, Camino de Vera s/n, 46022 Valencia, Spain; 2 Department of Plant Biology and Soil Science, Faculty of Biology, Universidad de Vigo, Campus Lagoas-Marcosendre, 36310 Vigo, Spain; 3 Mediterranean Agroforestry Institute (IAM), Universitat Politècnica de València, Camino de Vera s/n, 46022 Valencia, Spain

**Keywords:** eggplant, drought, stress tolerance, oxidative stress, interspecific hybrids, stress biomarkers

## Abstract

In a climate change scenario, crop tolerance to drought must be urgently improved, as it represents an increasingly critical stress reducing agricultural yields worldwide. Although most crops are relatively sensitive to water stress, many of their wild relatives are more tolerant and may be used to improve drought tolerance in our crops. In this study, the response to drought of eggplant (*Solanum melongena*), its close wild relatives *S. insanum* and *S. incanum* and their interspecific hybrids with *S. melongena* was assessed. The plants were subjected to two treatments for 18 days: control, with irrigation every four days, and drought, with complete interruption of irrigation. Morphological and biomass traits were measured, and physiological and biochemical responses were analysed using stress biomarkers such as proline, flavonoids, and total phenolic compounds. Oxidative stress was quantified by measuring malondialdehyde (MDA) content. As a result of the drought treatment, plant development and tissue water content were seriously affected. Generally, water deficit also caused significant increases in MDA, proline, flavonoids, and total phenolics compounds. Our results comparing parental accessions reveal a better response to drought in one of the *S. insanum* accessions. The hybrid between *S. melongena* and *S. incanum* displayed a better response than the other hybrids and even its parents. The results obtained here might be helpful for future eggplant breeding programmes aimed at improving drought tolerance.

## 1. Introduction

Eggplant (*Solanum melongena* L.) is an important and widespread vegetable crop, mainly in tropical and subtropical areas. Taxonomically, it is part of the “spiny” group of *Solanum* (subgenus *Leptostemonum* Bitter) [1]. It is a healthy vegetable for its fibre content, micronutrients, and bioactive compounds [2]. Specifically, it stands out for its high phenolic compounds content and, therefore, strong antioxidant capacity [3]. Eggplant is one of the crops whose global production increased the most between 2010 and 2020, with an increase of 26% from 44 × 10^6^ t to 56.6 × 10^6^ t [4]. Despite its economic importance, compared to other important vegetable crops, fewer efforts have been devoted to its genetic improvement [5]. Although eggplant is moderately tolerant to drought compared to other Solanaceae [6], water deficit can cause severe yield losses and affect fruit quality [7,8]; therefore, increasing its drought tolerance is a significant breeding objective in a scenario of climate change [9].

Cultivated species usually contain only a fraction of the genetic diversity found in their wild relatives [10,11]. Eggplant wild relatives are commonly exploited for eggplant improvement, but genetic improvement strategies rely on the information on the genetic basis of the inheritance of target traits [12]. Accordingly, some species have a high potential for improving and expanding the eggplant genetic base [13,14]. Obtaining more eggplant drought-tolerant cultivars depends on the identification of germplasm of interest and the introgression of relevant genes. Both steps require efficient, fast, and preferably inexpensive methods [15,16,17]. With the development of new generations of cultivars with introgressed genes providing tolerance, higher yields could ultimately be obtained in the face of climate change effects. Despite its advantages, interspecific hybridisation as a breeding method in eggplant is not very common. Currently, no known commercial cultivars of eggplant contain introgressions of wild species [18], contrary to what occurs in other crops such as tomato, wheat, or chickpea [19,20].

Crop wild relatives can be assigned to specific gene pools, in which species are included depending on their ability to cross and phylogenetic relationships with a cultivar [21]. The primary genepool of eggplant consists of the species *Solanum insanum* L. [13,22], as hybridisation with eggplant is highly efficient, and hybrids are very fertile. *Solanum insanum* is the closest wild species to eggplant and probably the ancestor of the cultivated species [23,24]. It can be found naturally in Southeast Asia, Madagascar and Mauritius [1]. As it has a good performance under drought conditions, this species is interesting for developing new eggplant varieties or rootstocks with increased drought tolerance. The secondary genepool comprises about 50 species from Africa and Southeast Asia [25,26] whose success in hybridisation with eggplant depends on the species and direction of crossing [14]. One of these species is *S. incanum* L., of particular interest in this study because it grows in desert environments in North Africa and the Middle East [1,22]. Given the adaptation of these two wild species to drought conditions, their utilisation in eggplant breeding may result in the development of a new generation of cultivars adapted to climate change challenges [18]. A recent screening of nine different eggplant wild relatives has revealed a high variability amongst wild species in their response to drought [27]. The fact that interspecific hybrids with the primary genepool species *S. insanum* are intermediate or close to eggplant for many traits may facilitate the use of this species in introgression breeding. Although *S. insanum* has not been identified as a much better performer to drought than *S. melongena,* it has a different response which can lead to the complementation of genes and improvement of the character [27]. Interspecific hybrids of eggplant usually are highly vigorous [28]; therefore, they may be directly exploited as drought-tolerant rootstocks. Regardless of their ability to tolerate abiotic stresses, plants possess mechanisms to respond to their effects. The capability of plants to respond to abiotic stress is associated with their plasticity and the adaptability of plant traits to the fluctuating conditions of water availability [29]. A plant subjected to stresses must redirect its resources to the activation of defence mechanisms. As a result, growth can be drastically reduced [30]. Adverse environmental conditions often cause oxidative stress in plants, as a direct or secondary effect. Therefore, plants respond activating antioxidant systems, both enzymatic and non-enzymatic [31]. Phenolic compounds are responsible for diverse plant functions, such as structural (lignin) or hormonal roles [32,33], but are also potent antioxidants. Many phenolic compounds, such as flavonoids, can neutralise the effect of reactive oxygen species (ROS) [34,35]. ROS are chemical compounds including free radicals such as superoxide (O_2_^−^), hydroxyl (OH^−^) and perhydroxyl (O_2_H^−^) radicals and other molecules such as hydrogen peroxide (H_2_O_2_) or ozone (O_3_) [36]. Their cellular concentrations increase under stress conditions, leading to deleterious effects on the cell. ROS affects the stability and permeability of cell membranes through lipid peroxidation. They also oxidise amino acid residues affecting protein activity, and cause mutations in DNA by reacting with nitrogenous bases [32,37]. Malondialdehyde (MDA), a reactive aldehyde product of membrane lipid peroxidation, is a well-known biomarker of oxidative stress [38,39]. Peroxidation of membrane lipids results in the loss of membrane fluidity, alteration of the membrane potential, increased permeability to H^+^ and other ions and, finally, cell rupture [40].

Proline, which plays a key role in stress responses, is an amino acid involved in osmotic adjustment, neutralisation of ROS, and protection of membrane stability. Proline accumulates in the cytosol as a response to drought and salinity, contributing to osmotic adjustment [41]. The accumulation of this osmolyte in plants subjected to water and salt stress is common in stress-tolerant and stress-susceptible species [42]. A relatively higher increase in proline is not always related to a higher level of stress tolerance. Although, in general, proline accumulation has been positively correlated with stress tolerance [43,44,45], in some species or cultivars, no correlation was found [46], or even a negative correlation was observed; that is, under the same stress conditions higher proline contents were measured in less tolerant genotypes [47,48].

The primary aim of this work was to evaluate the potential of interspecific hybrids of *Solanum melongena* with close wild relatives for the improvement of cultivated eggplant drought tolerance, emphasising the biochemical mechanisms involved. Therefore, the relative drought tolerance of cultivated eggplant (*S. melongena*), its close wild relatives *S. insanum* and *S. incanum* and their interspecific hybrids were analysed during vegetative growth under controlled greenhouse conditions. Biochemical analyses of proline, MDA, phenols, and flavonoids were performed in the parental species and their hybrids to better understand the responses to stress in these species. Additionally, this study may allow the identification of biomarkers associated with water stress for their subsequent use in a rapid assessment of drought tolerance in eggplant wild relatives, interspecific hybrids, and introgression breeding materials.

## 2. Results

### 2.1. Substrate Moisture during the Water Stress Treatments

**Figure 1:**
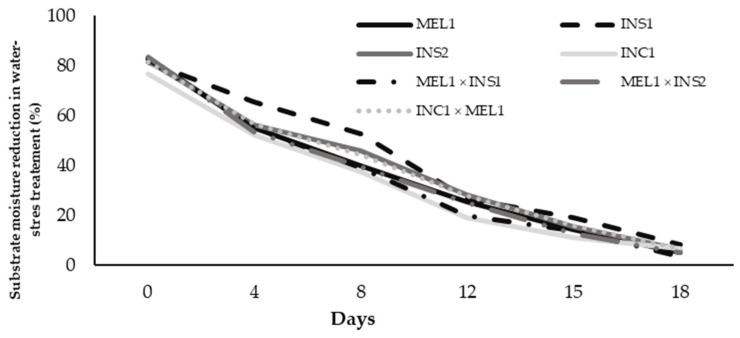
Reduction of substrate moisture in the water stress treatment measured by a WET-2 sensor in *Solanum melongena* (MEL1), *S. insanum* (INS1 and INS2), *S. incanum* (INS1) and their hybrids (MEL1 × INS1, MEL1 × INS2 and INC1 × MEL1). Each plotted value is the mean of the water content of five pots; each of these values, in turn, is the average of three independent measurements per pot.

During the 18 days of treatment, the control plants were watered to maintain the substrate’s moisture in a range of approximately 60–80%. Meanwhile, in the drought treatment, the plants did not receive irrigation, and the substrate water content decreased progressively to around 6%. The evolution of the substrate moisture of each material subjected to drought treatment is shown in Figure 1.

### 2.2. Growth Response of S. melongena, S. insanum and S. incanum during Water Stress

At the beginning of the treatments (t0), plants of the four parent accessions included in the study had an average height of 13.9 cm, stem diameter of 0.49 cm and 6–8 expanded leaves. Water stress reduced all growth parameters compared to the control: average reductions of 47.5% in total fresh weight, 18.9% in dry biomass, 16.2% in plant height, and 45.5% in leaf area were observed in the four genotype accessions evaluated. However, as indicated in the ANOVA (Table 1), quantitative differences between accessions were detected.

**Figure 2:**
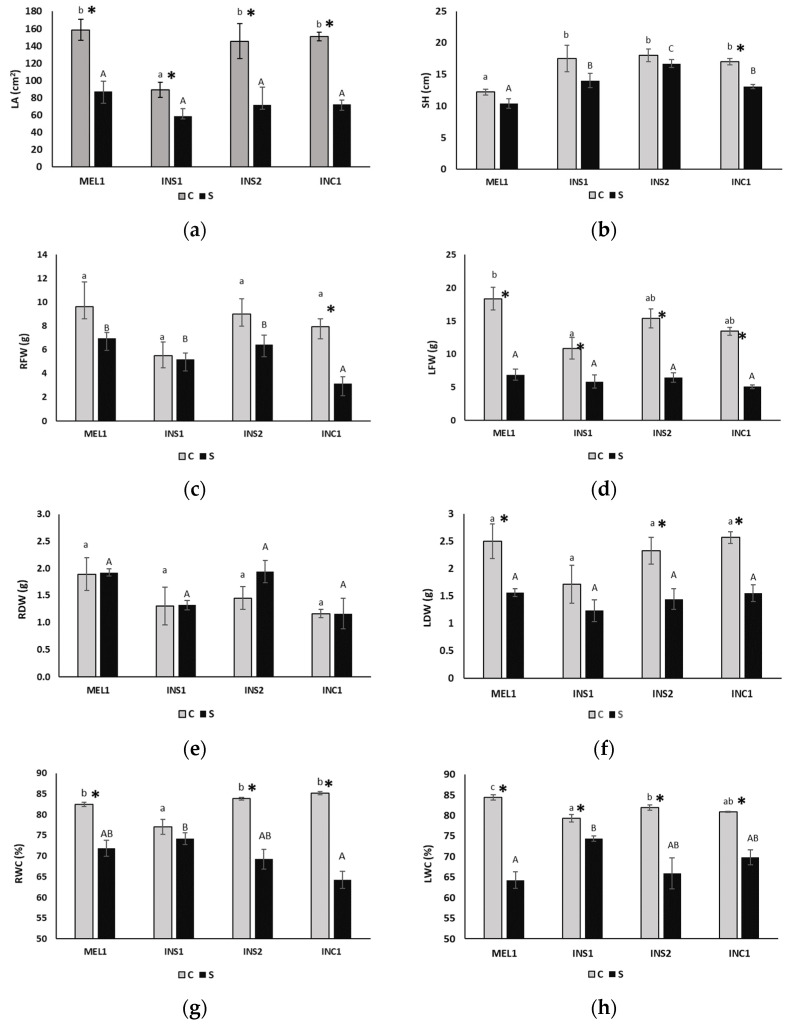
Variations in leaf area (**a**), stem height (**b**), root fresh weight (**c**), leaves fresh weight (**d**), root dry weight (**e**), leaves dry weight (**f**), root water content (**g**), leaves water content (**h**) between control and water-stressed plants of the indicated genotypes, after 18 days of treatment. Accessions are defined in Table 1. Bars represent means (*n* = 5) with standard errors. Different letters (lowercase for control plants and uppercase for water-stressed plants) indicate significant differences between genotypes according to the multiple range test with the Student-Newman-Keuls method at a *p*-value < 0.05. The asterisk indicates significant differences between the control and drought treatments, for each genotype, according to the *p*-value of the ANOVA F-test.

The drought treatment induced significant changes in the morphometric parameters evaluated, except for the dry weight of roots (RDW). Multifactorial ANOVA also revealed differences between genotypes for all parameters, except root water content (RWC), leaf dry weight (LDW) and leaf water content (LWC). The interaction between the genotype and the treatment was significant only for the water content of roots (RWC) and leaves (LWC). All genotypes were affected similarly by drought except for moisture parameters, where significant interactions were detected. Although all genotypes showed a significant decrease in leaf area under drought conditions (Figure 2a), the reduction percentage in INS1 was smaller, 34% with respect to the control. This could be because INS1 has a smaller leaf size; consequently, plants do not need to reduce the surface area available for transpiration. As for stem height, the treatment time did not allow significant changes to be observed, except in INC1 (Figure 2b). The impact of the treatment on growth can be best estimated by the differences in the fresh and dry weights of the different plant parts, as this provides information on biomass generation. The differences observed in roots were not statistically significant, except for the fresh weight (RFW) of INC1 (Figure 2c). INS2 was able to increase the mean root dry weight under drought conditions, although the differences with the control were not statistically significant, probably due to the short duration of the experiment. On the contrary, these differences were significant in leaves, except for INS1, whose leaf fresh and dry weights remained similar despite the drought effect (Figure 2d,f). The most significant differences were detected in fresh weight in MEL1, where the reduction was 59.5%, and in dry weight in INC1, where the weight was reduced by 39.8%. Water deficit caused a significant decrease, although not uniform, in water content in all accessions except for INS1 (Figure 2g,h). It is noteworthy that accession INS1 had a smaller reduction in water content than the others, both in the aerial part and in the root. It can also be observed how the water content of the leaves in all plants subjected to drought had a similar value. Other parameters, such as stem diameter or root length, did not differ significantly in response to the drought treatment (data not shown).

### 2.3. Biochemical Parameters Quantified in Parental Accessions

The analysis of biochemical parameters by multifactorial ANOVA (Table 2) revealed highly significant differences in the effect of genotype and treatment in malondialdehyde (MDA), total phenolic compounds (TPC), and total flavonoids (TF). All the evaluated species showed an increase in the biochemical compounds analysed under drought conditions with respect to the control, although with quantitative differences between them.

**Figure 3:**
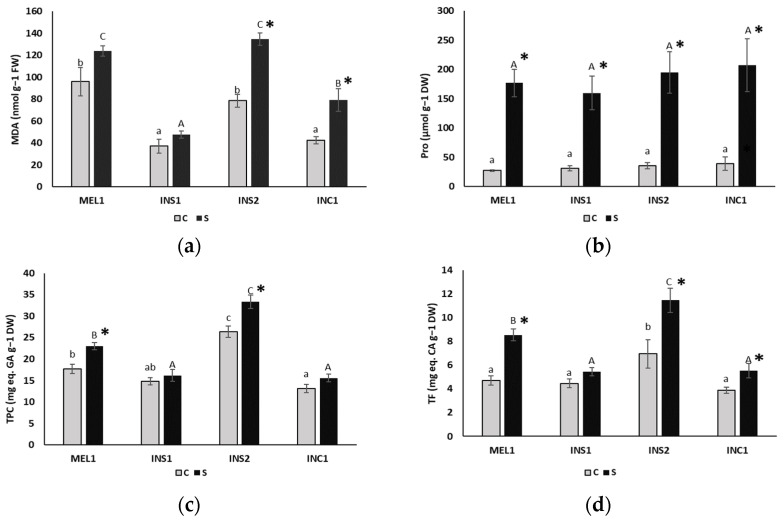
Variations in leaf MDA (**a**), proline (**b**), total phenolic compounds (**c**) and flavonoids (**d**) contents in plants of the indicated genotypes, after 18 days of control and water stress treatments. Accessions are defined in Table 1. Bars represent means (*n* = 5) with standard errors. Different letters (lower case for control plants and upper case for water-stressed plants) indicate significant differences between genotypes, according to the multiple range test with the Student–Newman–Keuls method at a *p*-value < 0.05. The asterisk indicates significant differences between the control and drought treatments, for each genotype, according to the *p*-value of the ANOVA F-test.

Due to the severe drought treatment, the MDA content increased 1.5-fold, on average, with respect to the control in INS2 and INC1, changing significantly in only half of the genotypes evaluated (Figure 3a). INS2 was the accession with the highest MDA content under water stress conditions, 134.6 nmol g^−1^ FW, thus showing a higher level of oxidative stress. It should be noted that relatively high MDA concentrations were also measured in non-stressed plants of MEL1 and INS2 (Figure 3b).

The proline content in control plants varied among species in a range of 10–38 μmol g^−1^ DW (Figure 3b) and increased in all cases in response to the stress treatment, reaching values between 81 and 208 μmol g^−1^ DW. This represents an average increase of 5.6-fold in proline in the water-stressed plants with respect to the non-stressed plants, a statistically significant difference in all cases. However, due to the high values of the standard deviation of the data, the differences between genotypes were not significant, under control or stress conditions (Figure 3b).

Phenolic compounds also increased in water-stressed plants, about 1.2-fold on average. Specifically, in the analysis of flavonoids, the greatest increase over the control, 1.6-fold, was observed in INS2. In most evaluated genotypes, significant differences between treatments were detected, except for TPC or TF in the INS1 accession, or for TPC in INC1. The phenolic and flavonoid content of INS2 was significantly different from the others, both in control and in water-stressed plants (Figure 3c,d).

### 2.4. Comparison of the Response of the Hybrids with Respect to Their Parents

**Figure 4:**
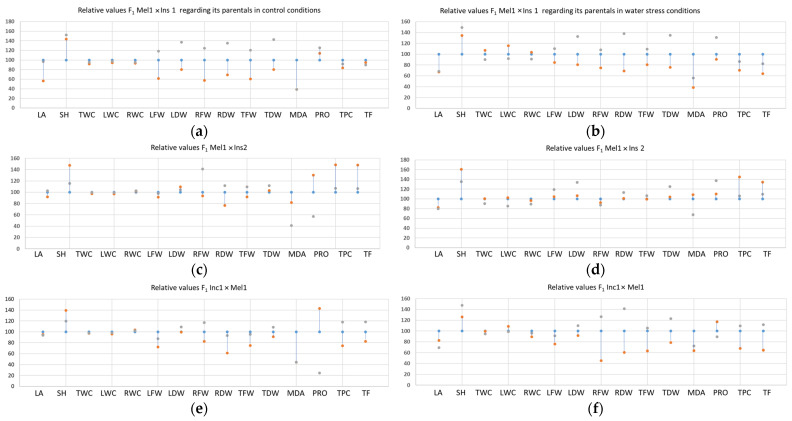
F1 relative values of MEL1 × INS1 in control (**a**) and under water stress (**b**); MEL1 × INS2 in control (**c**) and under water stress (**d**); INC1 × MEL1 in control (**e**) and under water stress (**f**). For each parameter, the MEL1 value was taken as a reference (100%), and the other values were referred to it. To clearly visualise superdominant or transgressive effects, a blue line has been drawn connecting the two parents. Abbreviations: leaf area (LA), stem height (SH), total water content (TWC), leaf water content (LWC), root water content (RWC), leaf fresh weight (LFW), leaf dry weight (LDW); root fresh weight (RFW), root dry weight (RDW), total fresh weight (TFW), total dry weight (TDW), foliar malondialdehyde (MDA), foliar proline (PRO), total foliar phenolic compounds (TPC), total foliar flavonoids (TF). Grey circles were used for hybrids, orange for the wild relatives and blue for the cultivated eggplant MEL1.

The three hybrids evaluated were also, as their parents, affected by drought stress. To compare their behaviour, heterosis, F_1_/Pm ratio and the potence ratio were calculated (Table 3 and Figure 4).

The MEL1 × INS1 hybrid deviated from the predicted value of the mean parental for most parameters. This hybrid showed significant heterotic effects for both root and aerial part weight parameters and stem height in control conditions. Interestingly, under drought conditions, this heterotic effect was less intense. Heterotic effects were also detected for proline and MDA contents but not for total phenolics or flavonoids, which showed an intermediate inheritance. Considering the potence ratio in the MEL1 × INS1 cross, superdominance was detected for almost all the biometrical parameters and the proline level in both control and water deficit conditions, so that the hybrid had higher biomass and proline content than the best parental (Figure 4). MDA content, on the contrary, showed dominance towards the *S. insanum* parental.

The other hybrid of *S. melongena* with *S. insanum*, MEL1 × INS2, in general, did not perform much better than the average of its parents for leaf area and stem height. However, it showed heterotic and superdominant effects for some biometric parameters such as RFW under control conditions, or root and leaf dry weight under stress conditions. Remarkably, this hybrid also showed heterotic effects for biochemical parameters, such as superdominance for MDA contents, indicating that it is less stressed than any of the parents in both tested conditions. In addition, it displayed superdominance for Pro values showing lower proline contents than any of the parents under control conditions but higher concentrations of the osmolyte than the parents in stressed plants.

The hybrid INC1 × MEL1 was not as heterotic as MEL1 × INS1 under control conditions but showed heterotic effects under stress for most studied biometrical parameters. Potence ratio values indicated that characters such as stem height had additive inheritance in the absence of stress but superdominance under water deficit. Superdominance was also detected for RFW in both, stress and non-stress conditions, whereas only under drought for RDW. Contrarily to Mel1 × *S. insanum* hybrids, the INC1 × MEL1 hybrid displayed a heterotic response for all the analysed compounds, such as dominance for MDA contents towards the *S. incanum* parental in both tested conditions, superdominance for proline values, with lower accumulation than in any of the parents, and also for the accumulation of total phenolic compounds and total flavonoids.

### 2.5. Criteria for Assessing Plant Tolerance

**Figure 5:**
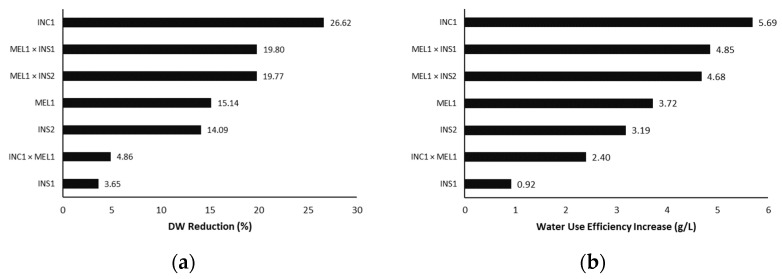
Percent reduction in dry biomass of total plant weight (**a**) and increase in water use efficiency (g/L) (**b**) in the water-stressed plants compared to their respective controls. The increase in water use efficiency was calculated as the increase in grams of dry biomass produced from t0 per litre of water consumed.

In this work, two simple calculations are proposed to rank plants according to their tolerance or sensitivity to stress: the reduction in dry weight and the increase in water use efficiency (Figure 5). The reduction in dry weight focuses on the reduction of biomass growth with respect to the control (Figure 5a). The increase in water use efficiency evaluates the capacity of each genotype to use the water provided to generate biomass under drought conditions (Figure 5b). It should be noted that this last parameter is defined as an increase and not a reduction because all the genotypes under water deficit conditions have improved this efficiency with respect to the control plants. Based on the dry weight reduction, INS1 showed the worst response of all genotypes, with a reduction of 33%. On the other hand, the INS2 accession had the lowest reduction of the parentals, with 14%. The *Solanum melongena* accession, MEL1, was surprisingly not the most affected by drought since the INC1 accession had a higher DW reduction than *S. melongena*. The INC1 × MEL1 and MEL1 × INS2 hybrids outstood by their low dry weight reduction under drought compared with the control, 3.65% and 4.86%, respectively, whereas MEL1 × INS1 showed an intermediate position between INS1 and MEL1. Regarding the increase in water use efficiency, MEL1 was the worst performer, followed by the *S. insanum* parentals. Interestingly, the hybrids performed better for this parameter than either parental. The same occurred with the hybrid INC1 × MEL1, which adapted to drought better than INC1.

## 3. Discussion

### 3.1. Growth and Biochemical Responses of Parental Species

Growth inhibition is a widely observed effect in plants under stress [49]. As a result of water stress, plants respond with morpho-anatomical, physiological, and biochemical adjustments aimed at counteracting the loss of water in an attempt to preserve their hydric status [29]. In our experiments, this effect was clearly observed after 18 days of stopping irrigation. The water deficit had a strong impact on most of the parameters analysed, generally higher than the genotype effect. The reduction of leaf area is a typical response of plants subjected to water stress, as they counteract the water limitation by reducing the transpiration area [50]. In our measurements, significant decreases in leaf area and leaf fresh and dry weight were observed in the water-stressed plants. On the contrary, the reduction in root fresh and dry weight was generally not significant. Maintaining or even increasing the absorption area is also a general response of plants to water deficit [51]. Similar findings were reported by Rahma [52] in genotypes related to *S. melongena*, where the dry weight of roots did not decrease significantly under water stress conditions. Interestingly, the biomass retention and root length were even higher in the hybrids.

Under drought conditions, there is usually a decrease in the number of leaves. This phenomenon is known as leaf senescence and is a regulated physiological process contributing to plant survival under adverse climatic conditions [53]. With this mechanism, nutrients are mobilised to other parts of the plant, and large water losses by transpiration are avoided [54], as described above for the reduction of leaf area. It was also observed that stem height remained similar in most genotypes despite the water deficit treatment, probably because the experiment did not last long enough to induce changes in this parameter. Regarding dry and fresh weight, INS1 is the accession with the lowest fresh weight reduction under drought conditions. A substantial relative water depletion was also detected in all species, although dehydration was not uniform in the selected genotypes. The INS1 accession appears to be more resistant to drought-induced dehydration. *Solanum insanum* is also recommended because it has a fruit size similar to some small-sized *S. melongena* cultivars, and this trait could be quickly recovered in backcrossing programmes [18]. Although eggplant is considered tolerant to mild water stress [55,56], the obtained results indicate that the complete absence of irrigation affects the *S. melongena* cultivar evaluated here. In fact, MEL1 turned out to be the accession with the lowest water use efficiency, showing that an increase in tolerance by using related wild species may be advisable for increasing cultivar performance.

Osmotic stress caused by drought induced the synthesis and accumulation of proline in the plants. Compatible solutes accumulated at high levels can contribute to tolerance without interfering with normal cellular metabolism [57]. Proline is one of the most common compatible solutes in plants, and its increase in response to water deficit has been observed in other species [46,58,59,60]. However, proline accumulation cannot always be positively correlated with tolerance [47]. All genotypes evaluated in this work showed very significant increases when subjected to water stress. In the study by Kurniawati [61], a significant increase in proline (10-fold over the control) was also observed in *S. melongena* plants under water deficit conditions, and we have previously reported similar results, especially in the most drought-tolerant eggplant cultivars [62]. The importance of this osmolyte for osmotic adjustment in *S. melongena* has been highlighted by other authors, for example by Tani et al. [63] and Sarker et al. [64], who found a correlation between higher proline levels under water deficit conditions and the maintenance of photosynthetic activity. On the other hand, the dispersion observed in the samples did not allow the detection of statistically significant differences between genotypes subjected to the drought treatment. However, the absolute proline concentrations reached after the treatment were high enough to have a relevant effect on osmotic adjustment, as reported in many other species [65]. Thus, although a positive correlation with tolerance cannot be directly established, the positive effect of this osmolyte in protecting against drought-induced dehydration cannot be dismissed.

When comparing the MDA and phenolic compounds levels, there is a rough correspondence between the level of drought-induced oxidative stress and the accumulation of antioxidant compounds. For example, in relation to other genotypes, a larger increase in MDA contents was detected in INS2, accompanied by a stronger induction of phenolics and flavonoid biosynthesis. In INS1, on the contrary, no significant differences between treatments were observed in TPC and TF contents, which corresponded to the measured low MDA levels. Therefore, this accession seems to be more resistant to drought-induced oxidative stress.

Phenolic compounds, particularly flavonoids, possess strong antioxidant and ROS-scavenging activities [66,67]. They are a good example of metabolites synthesised in plants in response to oxidative stress, especially when its effects are severe and the first line of defence against ROS, based on the activation of antioxidant enzymes, is overcome [68,69]. In most genotypes, the differences between control and water stress were statistically significant. The changes in flavonoids were qualitatively similar to those observed in phenolic compounds, as expected given that flavonoids represent the most numerous subgroup of phenolic compounds. The development of *Arabidopsis thaliana* mutants revealed that flavonoid accumulation is essential for improving drought tolerance in this model species [70]. According to Nisha et al. [71], flavonoids isolated from eggplant showed a potent free radical scavenging activity.

The flavonoid content was similar in the non-stressed plants of all accessions but not the concentration of total phenolic compounds. This suggests that the different genotypes must have a differential phenolics content, which may also be of interest if the antioxidant capacity of eggplant is to be increased. When analysing changes in phenolic compounds and flavonoid contents, it should be considered that these metabolites are responsible for many biological functions in the cell, as stated in the introduction [72,73]. These additional functions, unrelated to abiotic stress responses, may mask their specific effects on the stress tolerance mechanisms.

### 3.2. Evaluation of Hybrids’ Responses

Once a wild source of variability has been identified as potentially interesting, the next step in a breeding programme is to obtain interspecific hybrids between that wild species and a domesticated species. Usually, wild relatives carry undesirable traits, part of the linkage drag, that hampers breeding [74]; therefore, several steps of backcrosses are needed until achieving a good recovery of the agronomic characteristics of the cultivated crop [75]. On the other hand, it is well known that hybrid crops have superior yield performance compared with their parental lines, a phenomenon commonly defined as heterosis or hybrid vigour [76].

In this manuscript, the responses of three interspecific hybrids of eggplant with species of the primary and secondary gene pool have been tested. Under control conditions, the hybrid with higher hybrid vigour, understood as better growth than any of the parents, was MEL1 × INS1. Positive correlations between genetic distance and heterosis have been reported in several crops, although this is not a general rule for all traits or crops [77]. In fact, some authors suggest that heterosis can be better predicted only when the genetic distance is smaller than a certain threshold [78]. That would explain why the hybrid with *S. incanum* did not show higher heterosis than the hybrids with *S. insanum*.

Interestingly, all the hybrids tested showed higher performance under drought conditions than their corresponding parents. Strong heterotic effects and even transgressive or superdominant inheritance have also been reported in other crops under stress conditions [79,80]. Under stress, the plant tries to adapt to the new situation enhancing or silencing different sets of genes. In recent transcriptome studies, the importance of non-additive genes for the stress response has been determined and related to the heterotic responses [81].

In our experiments, heterosis was found not only for biometric traits but also for biochemical parameters. The results show a superdominant inheritance for MDA, where the hybrids were in general less stressed than the parents. The interpretation of the inheritance of proline levels is trickier as they were different depending on the hybrid and condition (control vs stress). This is not surprising in a context of different genetic backgrounds as proline metabolism is regulated in a complex way [82] and can be widely affected by the epistatic effects of many genes [83]. Transgressive values of proline have been found in hybrids of sunflower [41] and other crops [84] but their genetic regulation is still under study.

The inheritance for accumulation of phenolic compounds and flavonoids was different also depending on the genetic background: *S. insanum* hybrids showed intermediate inheritance, whereas the INC1 × MEL1 hybrid showed clear superdominance for both traits and both treatments. Again, heterotic effects for these compounds have been found in other crops [85,86]. The greater accumulation of flavonoids and other phenolic compounds appears to be a part of their improved tolerance mechanisms [27].

### 3.3. Future Perspectives

The development of ILs (introgression lines) using MEL1 as a recurrent parent could be the way forward for further studies. On the one hand, introgression lines would allow dissecting the genetic basis of the traits; on the other hand, eggplant varieties with drought tolerance would be generated by gene introgression. During introgression, each recombination event adds a possible permutation that should not be discarded, to identify rare cases of positive synergism. This has been exploited recently with coupling-uncoupling physiological effects obtaining large positive net gain in super-tolerant progeny by ideal complementation [87]. The fact that heterotic and transgressive effects have been found for different traits in *S. insanum* hybrids suggests that it is possible to find superior combinations within the produced lines even if the relative value of the donor was not much better than MEL1. In the same way, and considering its higher performance, the INC1 × MEL1 hybrid could also be used in a backcross programme to generate introgression lines with the aim of improving the tolerance of cultivated eggplant. In addition, INC1 × MEL1 and MEL1 × INS1 could be of interest for direct use as rootstocks because of their enhanced root system. Finally, some hybrids may even be of commercial interest. This may be the case for a hybrid with *S. insanum*, given that in Southeast Asia it is sometimes harvested from the wild [13].

Despite the limitations, the information provided in this manuscript will be useful in breeding projects where gene introgression into *S. melongena* is considered.

## 4. Materials and Methods

### 4.1. Plant Material

The plant material used in this work included a total of seven accessions and hybrids of eggplant and wild relatives: one cultivated eggplant accession (*S. melongena*), three accessions of two related wild species (*S. insanum* and *S. incanum*) and three interspecific hybrids between the *S. melongena* accession and the three accessions of wild relatives (Table 4). The seeds used were provided by the germplasm bank of the Institute for the Conservation and Improvement of Valencian Agrodiversity (COMAV, UPV, Valencia, Spain).

The cultivated eggplant accession (MEL1) originates from Côte d’Ivoire. Compared to other *S. melongena* accessions in the germplasm bank, MEL1 can produce multiparous inflorescences and many flowers, which is of interest to perform many crosses when used as the female parent. This accession is also an excellent recurrent parent to obtain interspecific hybrids with a degree of success higher than other *S. melongena* accessions [14,88]. The wild species accessions belong to the primary and secondary genepools of eggplant. Precisely, two of these accessions correspond to *S. insanum* (primary pool) with origin in Sri Lanka (INS1 and INS2) and one to *S. incanum* (secondary gene pool) from Israel (INC1) (Table 4).

The three interspecific hybrids were obtained by reciprocal crosses between cultivated eggplant and the wild species [14]. These hybrids are: MEL1 × INS1 (cross of *S. melongena* with *S. insanum*), MEL1 × INS2 (cross of *S. melongena* with another accession of *S. insanum*) and INC1 × MEL1 (cross of *S. incanum* with *S. melongena*) (Table 4).

For the germination of seeds, the protocol optimised by Ranil et al. [89] was followed, with slight modifications. Seeds were first soaked for 24 h in water, followed by another 24 h soaking in gibberellic acid solution at 500 ppm; after that, the seeds were transferred to Petri dishes with a KNO_3_ solution at 1000 ppm, used as a wetting agent. Then, the seeds were subjected to a heat shock for 24 h at 37 °C. After the heat shock, seeds were left to germinate in a climatic chamber under controlled conditions of 16 h light/8 h dark at 25 °C. Once germinated, seeds were sown in seedbeds and maintained under the same conditions in the growth room for two weeks. Subsequently, seedlings of homogeneous size were selected for transplanting into pots. When they were sufficiently grown, they were transferred to 1.3-L pots filled with 500 g of commercial substrate Huminsubstrate N3 (Klasmann-Deilmann, Geeste, Germany).

### 4.2. Experimental Design and Growth Conditions

Two treatments were applied to the plants: a normally watered control and a water stress treatment. At the beginning of the treatments, a subset of five plants from each of the seven selected genotypes was measured for non-destructive growth traits. Then, the remaining plants were irrigated with 250 mL of water to ensure that all pots were at a similar field capacity. For the duration of the experiment (18 days), the control plants were irrigated at 4-day intervals by supplying 250 mL of water per pot and per irrigation, whereas no irrigation was supplied to the plants subjected to drought treatment. During the experiment, the water content of the substrate was monitored periodically using a WET-2 sensor (Delta-T Devices, Cambridge, UK). This sensor allows a direct and in situ moisture determination in a non-destructive way. Mean values for each pot consisted of three measurements taken at different positions in the pot.

### 4.3. Measurement of Growth Parameters and Water Use Efficiency

Growth inhibition caused by stress was estimated by measuring various parameters. During the trial, plant development was monitored by measuring various morphological parameters once a week: stem height (SH, cm), number of leaves (Lno), considering only those that were more than 1 cm long, and stem diameter (Ø, mm). After 18 days of treatment, other destructive measurements were also taken. With the help of shears, each part of the plant was separated into root, stem and leaf material. The root of each plant was carefully cleaned using a brush to remove the substrate. The fresh weight of the aerial part of the plant, separating stem (SFW, g) and leaves (LFW, g) and the root fresh weight (RFW, g) was measured using an analytical balance. Using a ruler, the maximum root length (Lr, cm) was also determined. In addition, the area of the largest leaf of each plant (AF, cm^2^) was determined after scanning the leaves using the ImageJ software [90].

Once the fresh weight of the plant parts was measured, they were dried at 65 °C until a constant weight was reached. The water content of each plant part (WC) was determined using the following equation, where FW is the fresh weight, and DW is the dry weight.

WC (%) = [(FW − DW)/FW] × 100

The dry weight values were also used to calculate water use efficiency, as the increase in dry biomass generated during the treatment per litre of water supplied. This calculation was made for the plants subjected to drought and the control plants by subtracting the dry biomass from time 0 and assuming that during the 18 days of treatment, control plants received 1.25 L and the plants subjected to water stress 0.25 L—from the irrigation carried out only at the beginning of the treatments.

The water use efficiency parameter was calculated as:

WUE (g/L) = TDW/TWA

where TDW is the total dry weight of the plant and TWA is the total amount of water applied to each plant.

### 4.4. Biochemical Analyses

#### 4.4.1. Malondialdehyde (MDA) Determination

MDA contents were determined in methanol extracts, as previously described [91]. Fresh leaf material (ca. 150 mg) was extracted with 2 mL of 80% (*v*/*v*) methanol. Samples were shaken gently overnight and centrifuged to collect the supernatants. Methanol extracts were mixed with 0.5% (*w*/*v*) thiobarbituric acid (TBA) prepared in 20% (*w*/*v*) trichloroacetic acid (TCA), and then incubated for 20 min at 95 °C, cooled on ice and centrifuged at 13,300× *g* for 10 min at 4 °C. For each sample, a control containing the extract and TCA, but not TBA, was assayed in parallel. The absorbance of the supernatants was measured at 532 nm. The non-specific absorbance at 600 and 400 nm was subtracted, and the MDA concentration was calculated using the equations in [91]. MDA contents were expressed as nmol g^−1^ DW.

#### 4.4.2. Total Phenolic Compounds (TPC)

TPC contents were determined in the same extracts used for MDA quantification, as described by Singleton and Rossi [92], with some modifications [93]. The extracts were incubated with sodium carbonate and the Folin-Ciocalteu reagent for 90 min, at room temperature, in the dark, and the absorbance of the samples was then measured at 765 nm. Samples with known amounts of gallic acid (GA) were assayed in parallel to obtain a standard curve. TPC concentrations in the plant samples were expressed as ‘mg equivalent of GA g^−1^ DW’.

#### 4.4.3. Total Flavonoids (TF)

TF concentration in the leaf methanol extracts was determined as described by Zhishen et al. [94] by incubation with NaNO_2_—for the nitration of aromatic rings containing a catechol group—followed by reaction with AlCl_3_ at a basic pH. The product of the reaction was detected spectrophotometrically at 510 nm. TF contents in the plant material were expressed as equivalents of catechin, used as the standard (mg eq. C g^−1^ DW).

#### 4.4.4. Proline (Pro) Determination

Pro concentration was quantified according to the ninhydrin-acetic acid method [95]. Briefly, extracts were prepared by grinding the leaf material (50–100 mg) in 2 mL of a 3% (*w*/*v*) sulphosalicylic acid solution. The samples were mixed with acid ninhydrin, incubated for one h at 95 °C in a water bath, cooled to room temperature, and extracted with toluene. The absorbance of the organic phase was measured at 520 nm, using toluene as the blank. Reaction mixtures containing known Pro concentrations were run in parallel to obtain a standard curve. Leaf Pro contents were finally expressed in μmol g^−1^ DW.

### 4.5. Statistical Analysis

Statistical analysis was performed using Statgraphics Centurion XVII (Statpoint Technologies, Inc.; Warrenton, VA, USA). The data were processed using a multifactorial ANOVA, where genotype and treatment parameters were treated as fixed effects. The mean square of the effect of genotype, treatment, genotype × treatment interaction and residual effects were considered. Statistical differences between accessions subjected to the same treatment were analysed by ANOVA using the Student-Newman-Keuls multiple range test. The Student-Newman-Keuls procedure was used to evaluate which means are statistically different from each other. The statistical significance of the differences between the two treatments within each species was also analysed using the *p*-value of the ANOVA F-test.

Heterosis of the hybrids was calculated as:

Heterosis = 100 × [(F_1_ − PM)/PM]

Being F_1_ the hybrid values and PM the mean of both parentals (mean parent). The significance of the heterosis was tested by a Student’s *t* test, using the critical values for significance at *p* < 0.05, *p* < 0.01 and *p* < 0.001. Also the F_1_/Pm ratio was used to compare the hybrid with its parents. The potence ratio to was calculated as:

Pr = 100 × [(F_1_ − PM)/((Pmax − Pmin)/2)]

where Pmax and Pmin stand for the parental with higher or lower value respectively in each evaluated trait. Values of Pr equal to 0 indicate lack of dominance, Pr between −1 and 1 indicates partial dominance, Pr values equal to 1 indicate complete dominance and values of Pr higher than 1 or −1 indicate superdominance or transgressive inheritance [96,97].

## 5. Conclusions

The results presented here provide relevant information on the morphological and biochemical responses of eggplant and related wild species to water deficit. The drought treatment caused decreases in most morphological parameters except for the root, which maintained or even increased its biomass and thus the water absorption surface. INS1 showed the greatest tolerance to drought, as it stands out for being the accession with the lowest water loss. This genotype could be a good starting material for gene introgression in *S. melongena*. On the other hand, our findings indicated that *S. melongena* accession (MEL1) was not the most sensitive of all those investigated. Of the three hybrids evaluated (MEL1 × INS1, MEL1 × INS2 and INC1 × MEL1), INC1 × MEL1 was the most drought-tolerant as it had a better response than the other hybrids and its own parents. The hybrid MEL1 × INS1 showed hybrid vigour under favourable conditions (no water deficit), and therefore could be of interest as rootstock for non-stress conditions. Regarding the biochemical parameters analysed, relatively high levels of malondialdehyde (MDA) could be correlated with lower drought tolerance, and increased proline appeared to be a general protective response in all genotypes, contributing to osmotic adjustment. Oxidative stress induced the activation of phenolic compounds, including flavonoids, biosynthesis in most accessions. These results provide useful information for the design of a new generation of eggplant cultivars with increased drought tolerance.

## Figures and Tables

**Table 1:** Analysis of variance for growth parameters measured in control and water-stressed plants of parental genotypes. The values shown represent the mean square of the following parameters: root fresh weight (RFW), root dry weight (RDW), root water content (RWC), stem height (SH), leaf number (Lno), leaf area (LA), leaf fresh weight (LFW), leaf dry weight (LDW), and leaf water content (LWC).

Effects	gl	RFW	RDW	RWC	SH	Lno	LA	LFW	LDW	LWC
**MAIN**										
A: Genotype	3	20.97 *	1.08 ***	11.04 ^NS^	65.12 ***	12.33 **	3812 **	71.65 **	1.22 ^NS^	14.9 ^NS^
B: Treatment	1	62.70 **	0.16 ^NS^	1407 ***	64.85 ***	38.12 ***	37963 ***	1174 ***	12.17 ***	105 ***
**INTERACTION**										
AB	3	7.48 ^NS^	0.14^NS^	128.24 ***	3.89 ^NS^	1.63 ^NS^	1047 ^NS^	25.81 ^NS^	0.09 ^NS^	95.18 **
RESIDUALS	30	5.88	0.21	11.53	3.97	2.30	594	12.35	0.66	15.10

gl = degree of freedom, NS, *, ** and *** indicate not significant for a *p*-value > 0.05 and significant for *p* < 0.05, 0.01 and 0.001, respectively.

**Table 2:** Analysis of variance of biochemical assays. The columns show the mean square values of the analysis for malondialdehyde (MDA), total phenolic compounds (TPC), flavonoids (TF) and proline (Pro).

Effects	gl	MDA	TPC	TF	Pro
**MAIN**					
A: Genotype	3	9134 ***	483 ***	41.64 ***	1230 ^NS^
B: Treatment	1	9858 ***	150 ***	71.55 ***	184558 ***
**INTERACTION**					
AB	3	1257 *	15.42 ^NS^	6.64 ^NS^	2674 ^NS^
RESIDUALS	30		6.10	2.28	3622

gl = degree of freedom, NS, *, ** and *** indicate not significant for a *p*-value > 0.05 and significant for *p* < 0.05, 0.01 and 0.001, respectively.

**Table 3:** Analysis of the hybrids using their mean parents. For each analysed parameter, the mean of the five biological replicates of hybrids (F_1_), the mean parent (Pm), the F_1_/Pm ratio and Potence Ratio (P) are shown. One variable analysis of the heterosis by hypotheses test *t*, the asterisk indicates significant differences between the mean value of the F_1_ and the mean parental. This ratio is coloured according to its value using the colour relation shown below the table.

		MEL1 × INS1				MEL1 × INS2				INC1 × MEL1			
		F_1_	Pm	Ratio	P	F_1_	Pm	Ratio	P	F_1_	Pm	Ratio	P
**Leaf** **Area**	**C**	153.05	123.83	1.24	0.84	162.30	151.95	1.07	1.57	148.27	154.67	0.96	−1.66
	**S**	59.54	72.90	0.82	−0.94	69.40	79.51	0.87	−1.33	60.24	79.60	0.76 *	−2.57
**Stem** **Height**	**C**	18.60	14.85	1.25 *	1.42	14.10	15.10	0.93	−0.35	14.60	14.60	1.00	0
	**S**	15.50	12.20	1.27	1.83	14.10	13.55	1.04	0.18	15.36	11.75	1.31 *	2.67
**Total** **Water** **Content**	**C**	78.72	79.41	0.99	−0.20	82.32	81.78	1.01	0.52	80.89	81.59	0.99	−0.57
	**S**	60.33	69.46	0.87 *	−3.92	60.56	67.23	0.90	−62.46	63.56	67.02	0.95	−31.44
**Leaf ** **Water** **Content**	**C**	82.18	81.83	1.00	0.14	83.55	83.13	1.00	0.34	82.69	82.61	1.00	0.05
	**S**	59.09	69.34	0.85 *	−2.03	54.76	65.11	0.84	−12.61	63.55	67.06	0.95	−1.27
**Root ** **Water ** **Content**	**C**	78.69	79.99	0.98	−0.44	84.49	83.17	1.02	1.91	84.20	83.84	1.00	0.26
	**S**	65.43	73.87	0.89	−24.2	64.07	70.55	0.91	−12.61	69.00	68.09	1.01	0.24
**Leaf ** **Fresh** **Weight**	**C**	29.89	20.35	1.47 *	1.98	24.59	24.07	1.02	0.46	22.03	21.67	1.02	0.10
	**S**	11.22	9.41	1.19	2.34	12.16	10.41	1.17	7.82	9.28	8.95	1.04	0.27
**Leaf ** **Dry** **Weight**	**C**	5.17	3.39	1.52 *	4.79	3.93	3.94	1.00	0.06	4.10	3.76	1.09	54.74
	**S**	3.55	2.41	1.47 *	4.34	3.58	2.76	1.30	9.71	2.94	2.56	1.15	3.34
**Root ** **Fresh ** **Weight**	**C**	11.96	7.55	1.58 *	2.15	13.54	9.30	1.46 *	13.77	11.22	8.76	1.28 *	2.91
	**S**	7.50	6.07	1.24	1.64	6.06	6.68	0.91	−2.34	8.80	5.04	1.75 *	1.98
**Root ** **Dry ** **Weight**	**C**	2.55	1.60	1.60 *	3.24	2.11	1.67	1.26	2.00	1.76	1.49	1.19	0.65
	**S**	2.65	1.62	1.63	3.42	2.17	1.93	1.12	24.20	2.71	1.54	1.76 *	3.10
**Total ** **Fresh** **Weight**	**C**	41.85	29.99	1.40 *	2.03	38.13	33.37	1.14	3.35	33.25	30.44	1.09	0.65
	**S**	18.72	15.47	1.21	1.97	18.22	17.08	1.07	27.59	18.08	13.99	1.29 *	1.30
**Total ** **Dry ** **Weight**	**C**	7.72	4.87	1.59 *	5.25	6.04	5.49	1.10	7.20	5.87	5.16	1.14	2.84
	**S**	6.20	4.031	1.54 *	3.85	5.75	4.69	1.23	11.25	5.65	4.10	1.38 *	3.15
**Foliar ** **MDA**	**C**	37.33	66.43	0.56 *	−0.99	39.45	87.11	0.45 *	−5.48	42.43	66.16	0.64 *	−1.00
	**S**	69.29	85.76	0.81	−0.43	84.02	129.25	0.65 *	−8.50	89.36	101.44	0.88	−0.54
**Foliar ** **Proline**	**C**	33.90	28.91	1.17	2.65	15.42	31.58	0.49 *	−3.83	6.64	33.49	0.20 *	−4.50
	**S**	231.15	169.23	1.37	7.37	243.03	185.77	1.31	6.40	157.63	192.06	0.82	−2.26
**Foliar ** **Total ** **Phenol.**	**C**	16,28	16,28	1.00	0	18.98	22.23	0.85 *	−0.71	20.95	15.45	1.36 *	2.40
	**S**	19.91	19.58	1.02	0.097	24.34	28.17	0.86	−0.74	25.18	19.28	1.31 *	1.59
**Foliar ** **Total** **Flav.**	**C**	4.21	4.58	0.92	−3.05	5.00	5.82	0.86	−0.73	5.55	4.29	1.30 *	3.06
	**S**	7.06	7.00	1.01	0.04	9.38	10.01	0.94	−0.43	9.57	7.04	1.36 *	1.68
Note: 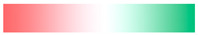													
					0.2		1		1.8				

**Table 4:** Information on the *Solanum* species analysed in this study.

Species	Accession	Germplasm Code	Origin	Genetic Pool	Hybrids
*S. melongena*	MEL1	BBS-118/B	Côte d’Ivoire		
*S. insanum*	INS1	SLKINS-1	Sri Lanka	Primary	MEL1 × INS1
*S. insanum*	INS2	SLKINS-2	Sri Lanka	Primary	MEL1 × INS2
*S. incanum*	INC1	MM664	Israel	Secondary	INC1 × MEL1

## Data Availability

Data are contained within the article.
